# Repeated romosozumab with short-term ibandronate sequencing in high-risk osteoporosis: a retrospective single-center cohort study

**DOI:** 10.1093/jbmrpl/ziag149

**Published:** 2026-08-27

**Authors:** Hiroyuki Maeda, Takahiro Maeda, Mutsuhiro Maeda

**Affiliations:** Tokyo Osteoporosis Center, Department of Orthopedics and Anesthesia, Yamamoto & Maeda Memorial Maeda Hospital, Higashikurume City, Tokyo 203-0054, Japan; Tokyo Osteoporosis Center, Department of Orthopedics and Anesthesia, Yamamoto & Maeda Memorial Maeda Hospital, Higashikurume City, Tokyo 203-0054, Japan; Tokyo Osteoporosis Center, Department of Orthopedics and Anesthesia, Yamamoto & Maeda Memorial Maeda Hospital, Higashikurume City, Tokyo 203-0054, Japan

**Keywords:** romosozumab, osteoporosis, high fracture risk, sequential therapy, treatment persistence

## Abstract

Long-term real-world evidence on repeated romosozumab treatment remains limited. This retrospective single-center cohort included 68 patients who initiated treatment between April 2020 and April 2026. Nineteen patients discontinued during the 5-yr observation period, and 49 completed follow-up and formed the longitudinal analytical cohort. Patients received romosozumab for 12 mo followed by oral ibandronate 100 mg once monthly for 3 mo before reassessment for another course. All patients received eldecalcitol 0.75 μg daily. Recorded LS and femoral young adult mean (YAM) values, bone turnover markers, numerical rating scale scores, persistence, fractures, and prespecified safety events were evaluated. Longitudinal outcomes were analyzed using linear mixed-effects models with a patient-level random intercept. Lumbar spine YAM increased from 73.3% at baseline to 92.9% at 5 yr, with a model-estimated change of 19.6 percentage points (95% CI, 17.9-21.3; *p* < .001). Femoral YAM increased from 66.2% to 76.1%, with a change of 9.9 percentage points (95% CI, 9.2-10.5; *p* < .001). Numerical rating scale scores decreased from 8.9 to 1.4, with an estimated change of −7.5 (95% CI, −7.9 to −7.1; *p* < .001). Interval-based treatment persistence was 72.1% at 5 yr (95% CI, 59.8%-81.2%). One recurrent vertebral fracture was recorded, whereas no cardiovascular events, hypocalcemia, osteonecrosis of the jaw, or atypical femoral fractures were recorded. Repeated courses were accompanied by longitudinal improvements in recorded skeletal measures and bone turnover markers. Because this was an uncontrolled retrospective study and patient-level baseline data for the 19 patients who discontinued were unavailable in the analytical dataset, the findings should be considered descriptive and hypothesis-generating.

## Graphical Abstract

**Graphical Abstract:**
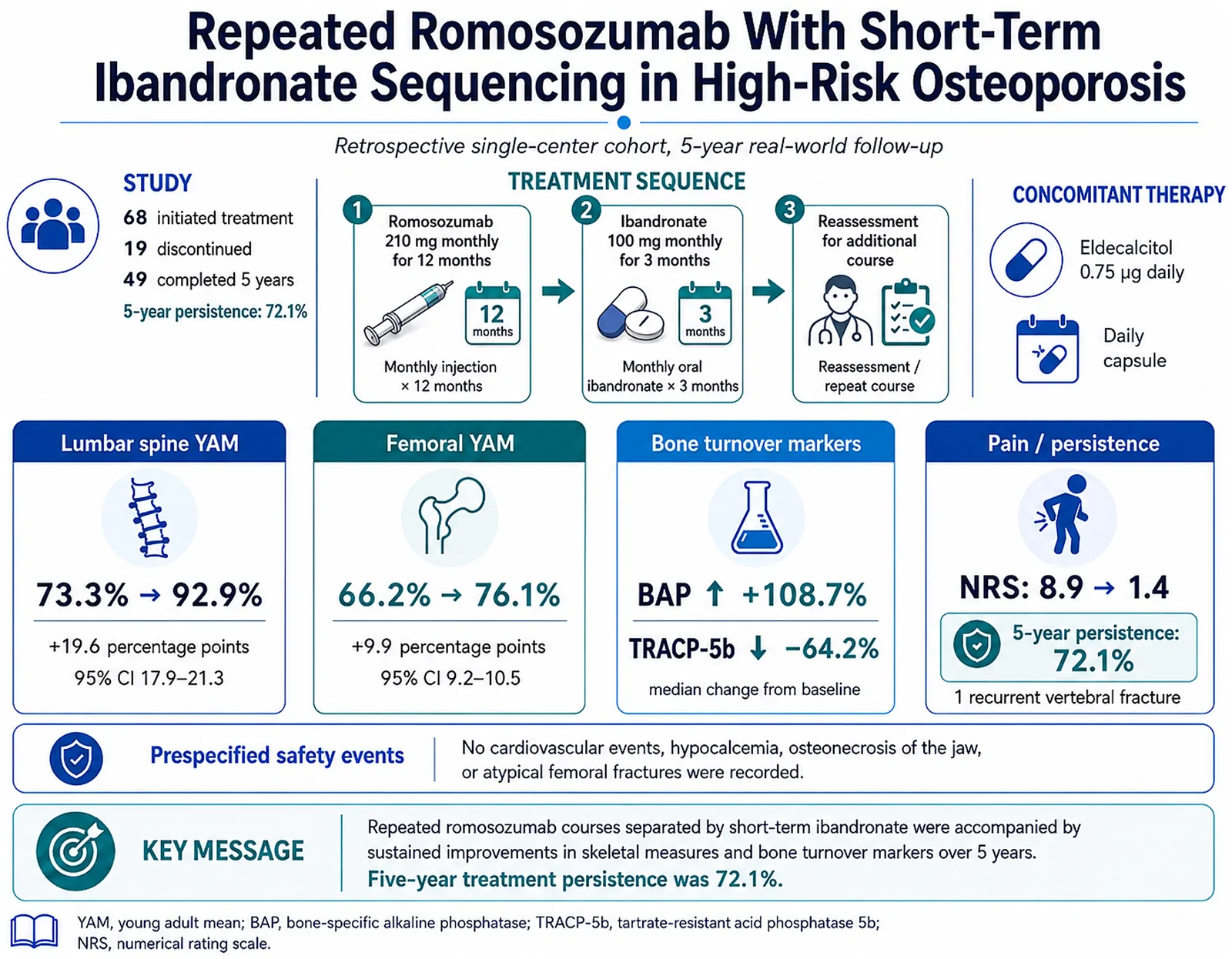

## Introduction

Romosozumab is an anti-sclerostin antibody with dual effects on bone formation and bone resorption.^1^ Although a single 12-mo course is supported by randomized trials,^2–4^ evidence regarding repeated courses and short-term antiresorptive sequencing in routine practice remains limited.^5–9^

The objective was to describe 5-yr changes in recorded young adult mean (YAM) values, bone turnover markers, pain scores, persistence, fractures, and prespecified safety events among patients receiving repeated romosozumab courses separated by short-term oral ibandronate therapy.

## Materials and methods

### Study design and cohort construction

**Figure 1:**
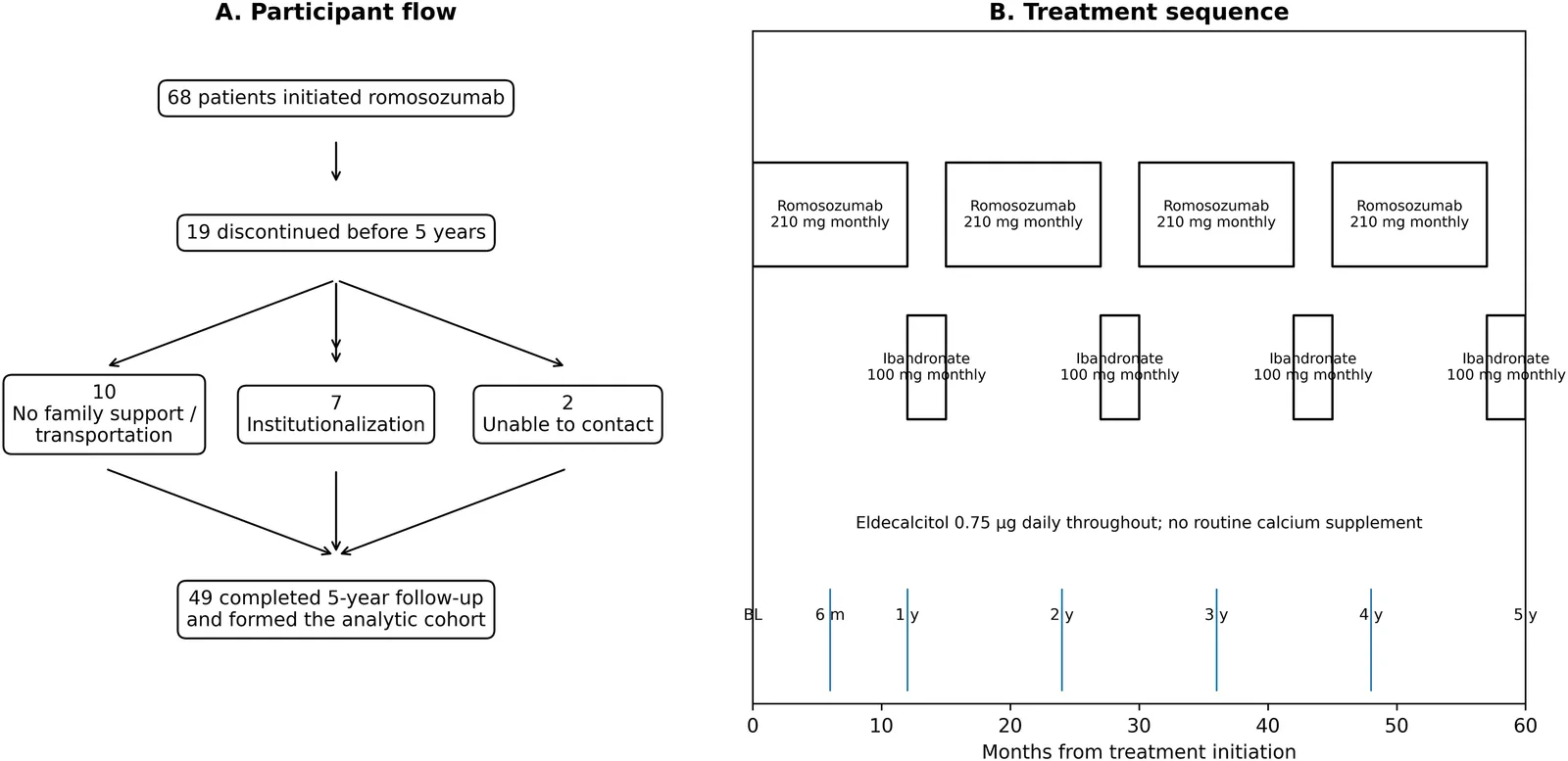
Participant flow and treatment sequence. Panel (A) shows 68 treatment initiators, 19 discontinuations, their reasons, and the 49-patient 5-yr analytical cohort. Panel (B) shows the planned sequence of 12 mo of monthly romosozumab followed by oral ibandronate 100 mg once monthly for 3 mo before reassessment. BL, baseline.

This retrospective single-center observational cohort was conducted between April 2020 and April 2026, with a maximum individual follow-up of 5 yr. Sixty-eight patients initiated romosozumab. Patients were consecutively included when treated at the study center, considered at high fracture risk according to Japanese criteria, and had sufficient records for longitudinal evaluation. High fracture risk was defined by at least one of the following: a BMD T-score of −2.5 or lower with one or more fragility fractures, a LS T-score below −3.3, two or more prevalent vertebral fractures, or a grade 3 prevalent vertebral fracture on semiquantitative assessment. These criteria correspond to the high-fracture-risk criteria referenced in the Japanese reimbursement notification for romosozumab.^10^ Nineteen patients discontinued: 10 because family assistance or transportation was unavailable, 7 because institutionalization prevented clinic attendance, and 2 because they could not be contacted. The remaining 49 patients completed 5 yr and formed the longitudinal analytical cohort. The participant flow and treatment sequence are shown in Figure 1.

### Treatment protocol

Romosozumab 210 mg was administered subcutaneously once monthly for 12 mo. After each 12-mo course, patients received oral ibandronate 100 mg once monthly for 3 mo before reassessment for an additional romosozumab course. All patients received eldecalcitol 0.75 μg orally once daily throughout treatment. No calcium supplement was routinely prescribed. No patient had documented prior osteoporosis pharmacotherapy or glucocorticoid use. Renal function remained within the institutional reference range without documented deterioration. Repeat treatment was considered only when the patient again met the same high-fracture-risk criteria. In accordance with the Japanese reimbursement notification, the reason for judging the patient to remain at high fracture risk and the name of the osteoporosis medication administered between romosozumab courses were documented in the medical claim; in this cohort, the intervening medication was ibandronate.^10^ This reimbursement framework was distinguished from evidence of comparative efficacy or a general recommendation for repeated treatment.

### Assessments

Areal BMD of the LS and proximal femur was measured by DXA using a Lunar Prodigy densitometer (GE HealthCare). Scans were analyzed using enCORE software, version 18. Quality assurance and calibration were performed each day before patient scanning using the manufacturer-supplied calibration block in accordance with the manufacturer’s instructions. Lumbar spine BMD was assessed using the mean of evaluable vertebrae from L1 to L4. Vertebrae affected by fracture, marked degenerative change, structural abnormality, or imaging artifact were excluded in accordance with International Society for Clinical Densitometry recommendations. A vertebra was also considered for exclusion when its T-score differed by more than 1.0 from that of an adjacent vertebra. At least 2 evaluable vertebrae were required.^11^ The least significant change was calculated as 2.77 times the site-specific precision error at our institution. Recorded YAM was retained as the primary skeletal outcome because direct DXA-exported absolute BMD values were unavailable in the analytical dataset. Bone-specific alkaline phosphatase (BAP), tartrate-resistant acid phosphatase 5b (TRACP-5b), and numerical rating scale (NRS) scores were recorded at baseline, 6 mo, and annually through 5 yr. Absolute BAP and TRACP-5b values are reported in μg/L and mU/dL, respectively. The same 49 patients contributed at all 7 time points.

### Persistence

Eight patients discontinued during year 1, 5 during year 2, 2 during year 3, 2 during year 4, and 2 during year 5. Exact discontinuation dates were unavailable; persistence was therefore estimated from annual intervals.

### Statistical analysis

Continuous variables are summarized as mean ± SD or median with IQR. Longitudinal outcomes were analyzed with linear mixed-effects models using categorical time as a fixed effect and patient as a random intercept. Changes from baseline are reported with 95% CIs. All 49 analyzed patients had complete observations, so the complete-case sensitivity analysis was identical to the primary analysis. A baseline comparison between completers and non-completers was not possible because patient-level baseline data for the 19 discontinuers were not retained. Annual persistence was estimated with an interval-based Kaplan–Meier approach. Event proportions use exact binomial 95% CIs.

## Results

Participant characteristics: Mean age was 84.8 ± 5.8 yr; 48 participants were women. Vertebral compression fracture was present in 31, proximal femoral fracture in 6, and another fragility fracture in 12 patients (Table 1).

**Table 1:** Baseline characteristics of the 49-patient longitudinal cohort.

**Characteristic**	**Value**
**Age, yr**	84.8 ± 5.8
**Female sex**	48 (98.0%)
**Male sex**	1 (2.0%)
**Height, cm**	150.0 ± 5.2
**Weight, kg**	50.4 ± 8.6
**BMI, kg/m** ^**2**^	22.4 ± 3.7
**Prevalent vertebral compression fracture**	31 (63.3%)
**Prevalent proximal femoral fracture**	6 (12.2%)
**Other fragility fracture**	12 (24.5%)
**Lumbar spine YAM, %**	73.3 ± 16.1
**Femoral YAM, %**	66.2 ± 12.6
**BAP, baseline μg/L**	7.9 (6.8-8.6)
**TRACP-5b, baseline mU/dL**	411 (249-649)
**NRS, baseline**	8.9 ± 1.1
**Prior osteoporosis medication**	None
**Glucocorticoid use**	None
**Renal function**	Within institutional reference range; no documented deterioration

Data are mean ± SD, *n* (%), or median (IQR).

Abbreviations: BAP, bone-specific alkaline phosphatase; NRS, numerical rating scale; TRACP-5b, tartrate-resistant acid phosphatase 5b; YAM, young adult mean.

**Table 2:** Mixed-effects model estimates, persistence, and clinical events.

**Outcome**	**Time**	**Estimate/events**	**95% CI**	***p*-value**
**Lumbar YAM change**	5 yr vs baseline	19.6 points	17.9 to 21.3	<.001
**Femoral YAM change**	5 yr vs baseline	9.9 points	9.2 to 10.5	<.001
**NRS change**	5 yr vs baseline	−7.5	−7.9 to −7.1	<.001
**Treatment persistence**	5 yr	72.1%	59.8% to 81.2%	—
**Recurrent vertebral fracture**	5 yr	1/49 (2.0%)	0.1% to 10.9%	—
**Cardiovascular event**	5 yr	0/49 (0%)	0% to 7.3%	—
**Hypocalcemia**	5 yr	0/49 (0%)	0% to 7.3%	—
**Osteonecrosis of the jaw**	5 yr	0/49 (0%)	0% to 7.3%	—
**Atypical femoral fracture**	5 yr	0/49 (0%)	0% to 7.3%	—

Abbreviations: NRS, numerical rating scale; YAM, young adult mean.

**Figure 2:**
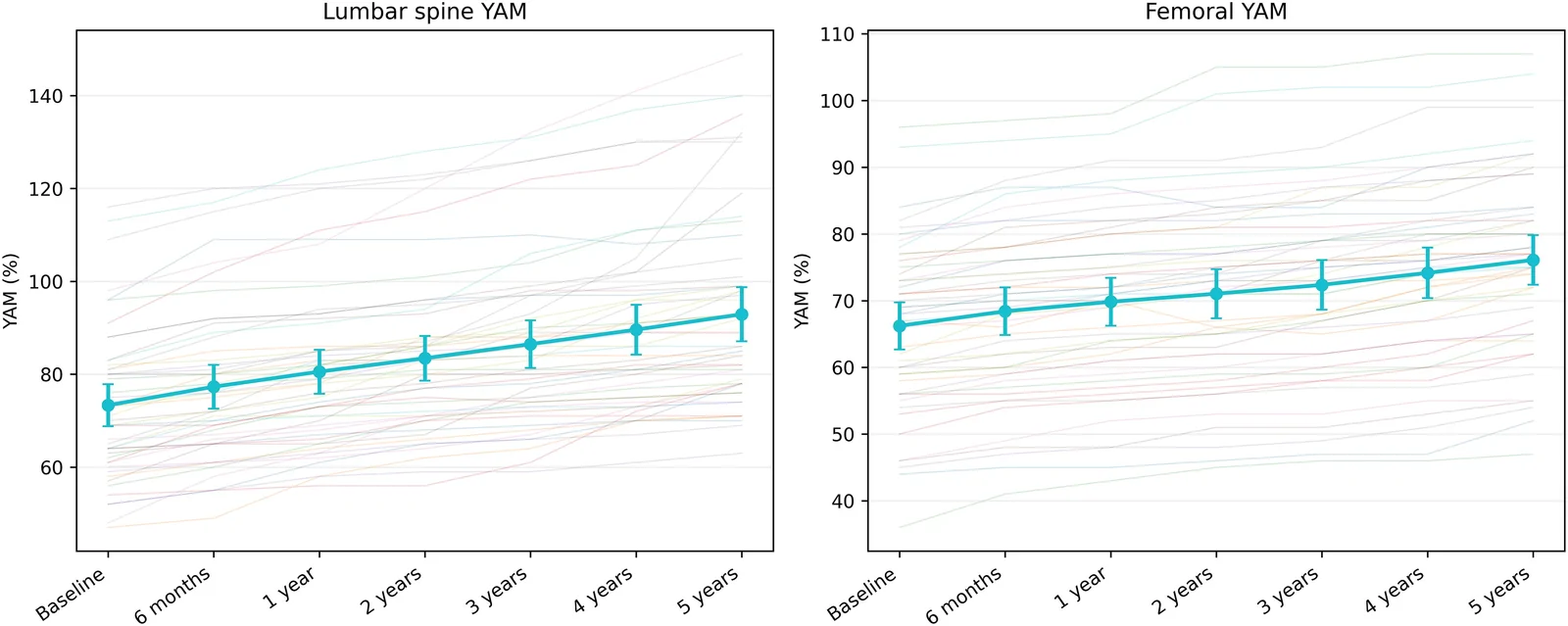
Individual LS and femoral young adult mean (YAM) trajectories. Thin lines represent individuals and the bold line represents the mean with 95% CIs.

Skeletal outcomes: Lumbar spine YAM increased from 73.3 ± 16.1% to 92.9 ± 20.8%. The mixed-model change was 19.6 points (95% CI, 17.9-21.3; *p* < .001). Femoral YAM increased from 66.2 ± 12.6% to 76.1 ± 13.2%; change 9.9 points (95% CI, 9.2-10.5; *p* < .001). Individual LS and femoral YAM trajectories are shown in Figure 2.

**Figure 3:**
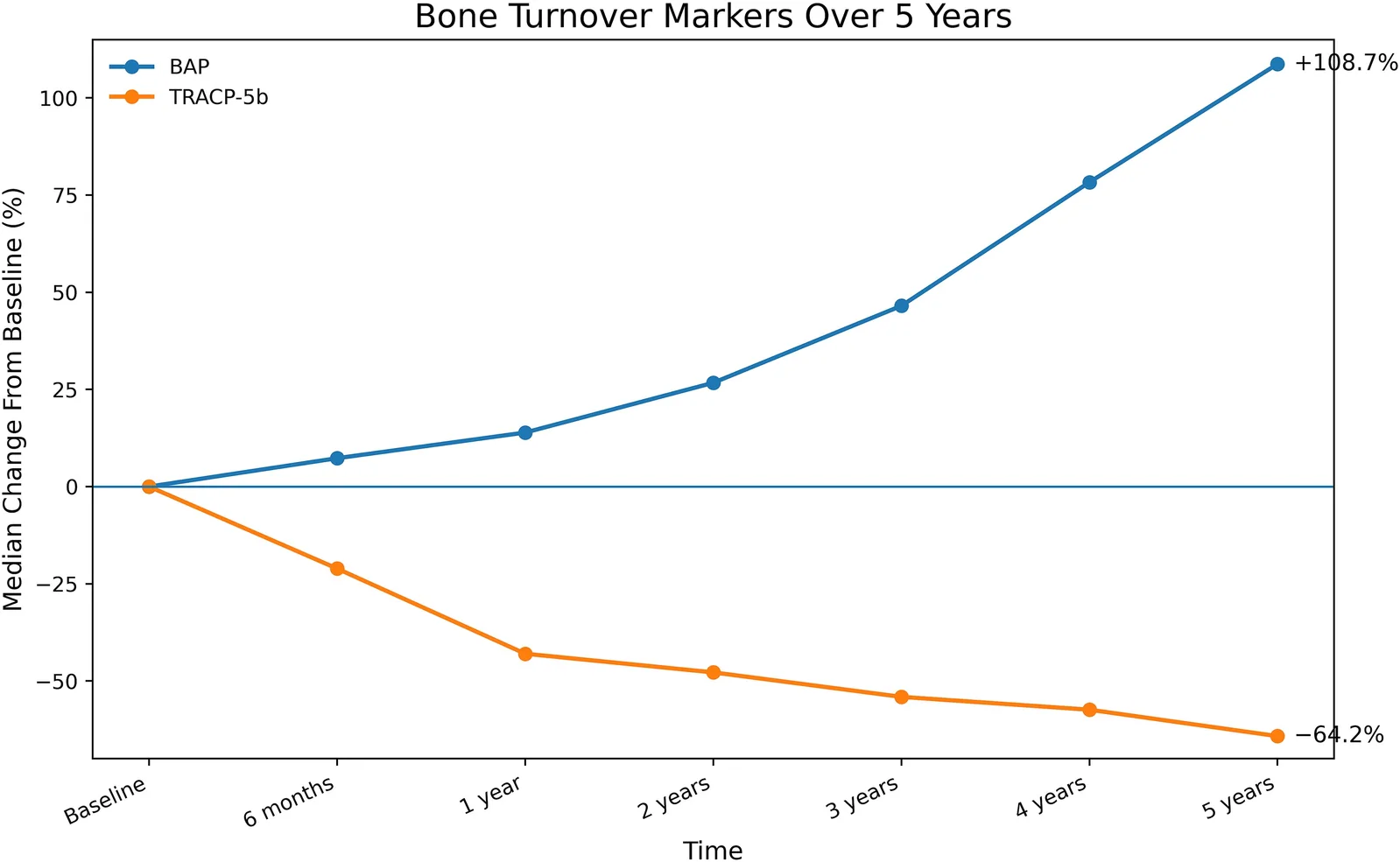
Bone turnover markers over 5 yr. Bone-specific alkaline phosphatase (BAP) and tartrate-resistant acid phosphatase 5b (TRACP-5b) are shown as median percent changes from baseline. BAP increased over time, reaching +108.7% at 5 yr, whereas TRACP-5b decreased to −64.2% at 5 yr. absolute median values with IQRs are reported in the results.

Bone turnover markers: Median BAP increased from 7.9 (6.8-8.6) μg/L to 17.2 (13.5-24.2)μg/L. Median TRACP-5b decreased from 411 (249-649)mU/dL to 160 (136-195)mU/dL. Bone turnover marker trajectories are shown in Figure 3.

Pain: Mean NRS decreased from 8.9 ± 1.1 to 1.4 ± 0.6; estimated change −7.5 (95% CI, −7.9 to −7.1; *p* < .001). This cannot be attributed specifically to romosozumab because analgesics, rehabilitation, natural healing, and regression to the mean were not controlled.

**Figure 4:**
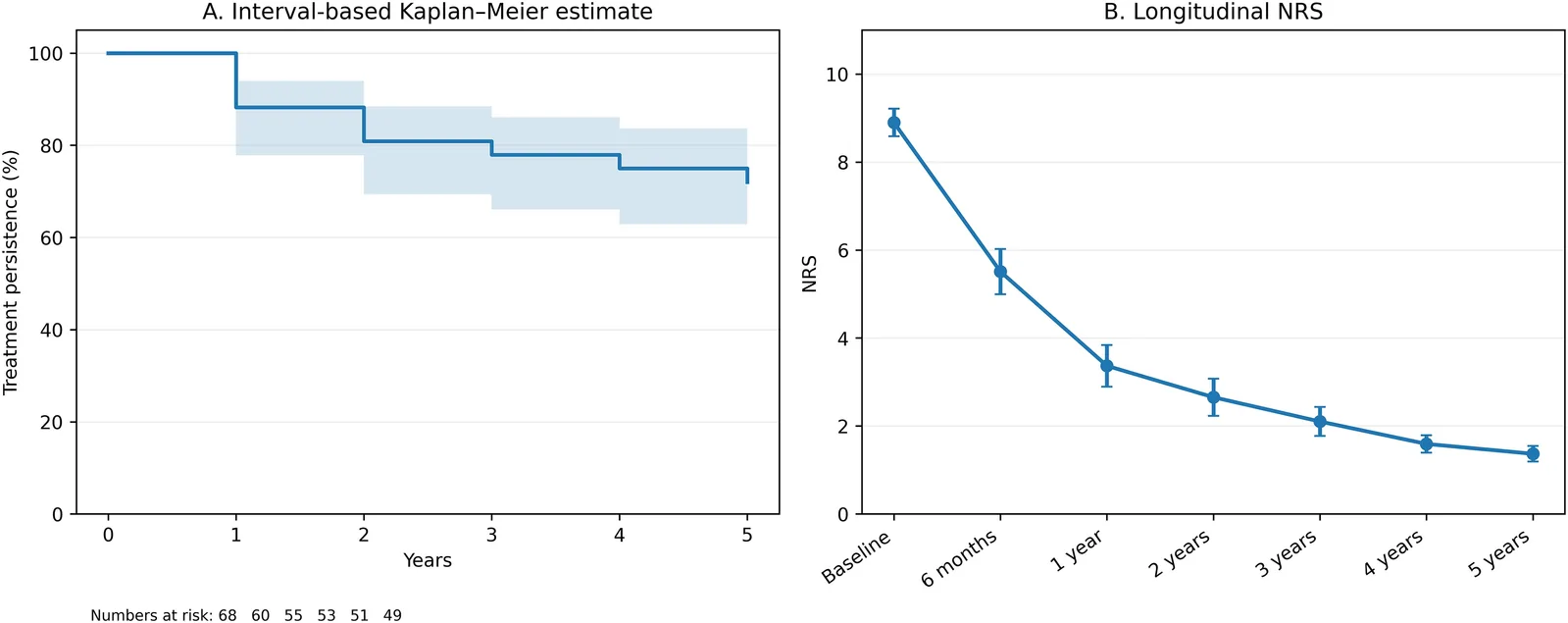
Treatment persistence and numerical rating scale (NRS) scores. Panel (A) shows interval-based persistence with 95% CIs and numbers at risk. Panel (B) shows mean NRS with 95% CIs.

Persistence: Interval-based persistence was 88.2%, 80.9%, 77.9%, 75.0%, and 72.1% at years 1-5. The 5-yr 95% CI was 59.8-81.2. Treatment persistence and NRS trajectories are shown in Figure 4.

Fractures and safety: One recurrent vertebral fracture was recorded (2.0%; exact 95% CI, 0.1-10.9). No cardiovascular events, hypocalcemia, osteonecrosis of the jaw, or atypical femoral fractures were recorded; for each zero-event outcome, the exact upper 95% confidence limit was 7.3%. These observations do not establish safety or fracture-prevention effectiveness.

## Discussion

Repeated romosozumab courses separated by short-term oral ibandronate were accompanied by progressive increases in recorded YAM and longitudinal changes in bone turnover markers. The mixed-effects model accounted for within-patient correlation. Complete-case sensitivity results were identical because all 49 analyzed patients had complete observations.

Recorded YAM is the defensible primary skeletal outcome. Multiplying YAM by a fixed constant does not recover direct DXA-exported BMD; calculated g/cm^2^ values were therefore removed.

The lumbar increase may have been influenced by degenerative change, vertebral deformity, or differences in evaluable vertebral levels. Vertebral-level exclusions, least significant change, scanner software version, and detailed calibration records were unavailable in the analytical dataset.

Pain reduction was observed, but treatment-specific analgesic effects cannot be inferred. Likewise, one recurrent fracture and no recorded major safety events cannot establish fracture-prevention effectiveness or safety.

Institutional education was provided to all patients, but its independent effect cannot be separated from selection, family support, clinic attendance, and other factors. Its effect remains a hypothesis for future comparative study.

This protocol represents intentional sequential therapy rather than administrative step therapy. The distinction is relevant to orthopedic osteoporosis practice and is discussed in relation to the recent review by Salman and colleagues.^9^

Japanese reimbursement rules explicitly describe the documentation required when romosozumab is re-administered after a completed 12-mo course: the clinician must record both the reason the patient is considered at high fracture risk and the name of the osteoporosis medication administered before re-treatment. Thus, re-treatment is contemplated within the Japanese reimbursement framework when these conditions are met. However, this administrative framework does not itself establish the effectiveness or safety of repeated courses, and the labeled regimen remains 210 mg once monthly for 12 mo.^10^

Limitations include the retrospective single-center design, lack of a comparator, selection of a 49-person complete longitudinal cohort from 68 treatment initiators, absence of patient-level baseline data for the 19 discontinuers, use of annual rather than exact discontinuation dates, and incomplete retention of direct absolute BMD data. Although repeat courses were performed only in patients who again met the specified high-fracture-risk criteria, were documented according to the Japanese reimbursement notification, and were covered by institutional ethical approval, this study does not establish the comparative effectiveness or safety of re-treatment. The administrative reimbursement provisions should not be interpreted as equivalent to prospective clinical evidence.

### Conclusion

Repeated courses were accompanied by longitudinal increases in recorded YAM and changes in bone turnover markers over 5 yr. Interval-based persistence was 72.1%. Because the study lacked a comparator, excluded 19 discontinuers from longitudinal analyses, and had incomplete DXA metadata, the findings are descriptive and hypothesis-generating.

## Data Availability

The data underlying this study are available from the corresponding author upon reasonable request, subject to institutional and privacy considerations.
